# 

**Published:** 1907-08

**Authors:** 


### The American Journal of Dental Science

The Official Organ of the Florida, Georgia and South Carolina State Dental Societies

**Third Series**

VOL. XXXVIII AUGUST, 1907 NO. 8

EDITORS

WM. GIRD BEECROFT, D. D. S., Madison, Wis. B. H. TEAGUE, D. D. S., Aiken, S. C.

Published on the 10th day of each month.
Subscription price $1.00 per year.
ENTERED AS SECOND-CLASS MATTER, MADISON, WIS.

Address all communications, both business and editorial, to
**The Dental Publishing Company, Madison, Wisconsin**

### MEETINGS.

Revised program for The Jamestown Dental Convention meeting to be held in Convention Hall, exposition grounds, Norfolk, Va., Sept. 10-11-12, 1907.

Officers—Hon. Pres. J. Y. Crawford, Nashville, Tenn.; Pres. V. E. Turner, Raleigh, N. C.; 1st Vice-Pres. B. Holly Smith, Baltimore, Md.; Secy-General, George S. Keesee, Richmond, Va.; Treas., Mark F. Finley, Washington, D. C.

Program, Tuesday, Sept. 10, 1907, 9:30 A. M.—Meeting called to order by Dr. Burton Lee Thorpe, St. Louis, Chairman Committee on Organization. Invocation—Rev. Dr. C.

L. Bane, Pastor Memorial M. E. Church, Norfolk, Va. Address of Welcome—Hon. Harry St. George Tucker, President Jamestown Exposition Co. Address of Welcome—Dr. Joseph W. Eggleston, Richmond, Va., in behalf of the profession of Virginia. Address of Welcome—Dr. Edward Eggleston, Richmond, President Virginia State Dental Association. Address of Welcome—Dr. W. G. Mason, Tampa, Fla., President Southern Branch N. D. A. Address of Welcome—Dr. J. Y. Crawford, Nashville, Tenn., in behalf of the profession of the south. Response to the Addresses of Welcome—Dr. J. D. Patterson, Kansas City, Mo. Address by the President—Dr. V. E. Turner, Raleigh, N. C.

Tuesday afternoon session, 2:30 P. M.—Clinics in Convention Hall—Dr. Clarence J. Grieves, Chairman, Baltimore, Md.

Tuesday evening, Sept. 10, 8:00 P. M.—Smoker at Inside Inn—Dr. B. Holly Smith, Chairman, Baltimore, Md.

Wednesday morning, Sept. 11, 9:30 A. M.—Illustrated Lecture—Dr. F. T. Van Woert, Brooklyn, N. Y., "Is the Cemented Filling, the Filling of the Future?" Discussion opened by Dr. Wm. K. Slater, Knoxville, Tenn., and Dr. Craig M. Work, Ottumwa, Iowa.

11:00 A. M.—Illustrated Lecture—Dr. Chas. L. Alexander, Charlotte, N. C., "Gold Inlays." Discussion opened by Dr. H. Herbert Johnson, Macon, Ga., and Dr. J. G. Fife, Dallas, Texas.

2:30 P. M.—Clinics in Convention Hall.

8:00 P. M.—Illustrated Lecture—Dr. R. Ottolengui, New York City, "The Purposes and Accomplishments of Modern Orthodontia." Discussion opened by Dr. W. O. Talbot, New Orleans, La., and Dr. Henry W. Morgan, Nashville, Tenn.

Thursday, 9:30 A. M.—Clinics in Convention Hall.

2:30 P. M.—Special Clinical Lecture and Demonstration by Dr. Wm. H. Taggart, Chicago, Ill., "Cast Gold Inlays, Bridges and Plates." Discussion opened by Dr. J. H. Lorenz, Atlanta, Ga., and Dr. L. E. Custer, Dayton, Ohio.

Adjournment, 8:00 P. M.—Entertainment given to members and guests of convention by Virginia State Dental Association under the chairmanship of the Society's President, Dr. Edward Eggleston, Richmond, Va.

A cordial invitation is extended to all ethical dentists to become members and attend this meeting.

All sessions are to be held in "The Convention Hall," at Exposition Grounds. Entrance to this hall is outside of the grounds, thus saving admission fee to enter it, however entrance to the grounds is possible without leaving the hall.

To expediate the work before the general sessions all resolutions, motions and routine business must first be submitted to the Committee on Organization who at the proper time will present it to the general body.

Committee on Organization—Burton Lee Thorpe, Chairman, 305 N. Grand Ave., St. Louis, Mo.; Thos. P. Hinman, Vice-chairman, Inmon Bldg., Atlanta, Ga.; F. W. Stiff, Treas., 600 E. Grace St., Richmond, Va.; R. H. Walker, Norfolk, Va.; J. E. Chace, Ocala, Fla.; Clarence J. Greives, Park & Madison Aves., Baltimore, Md.

H. Wood Campbell, Secy.,
Suffolk, Va.

